# Factors associated with utilization of emergency contraception among female students in Mizan-Tepi University, South West Ethiopia

**DOI:** 10.1186/s13104-015-1812-6

**Published:** 2015-12-24

**Authors:** Bisrat Zeleke Shiferaw, Bosena Tebeje Gashaw, Fekadu Yadassa Tesso

**Affiliations:** Department of Midwifery, Wolkite University College of Medicine and Health Sciences, PO Box 07, Wolkite, Ethiopia; Department of Nursing and Midwifery, Jimma University College of Public Health and Medical Sciences, PO Box 1355, Jimma, Ethiopia

**Keywords:** Emergency contraception utilization, Associated factors, Mizan-Tepi University

## Abstract

**Background:**

Unintended pregnancy poses a major health problem on female students in higher educations. One of the key interventions to reduce unintended pregnancy and unsafe abortion as outlined in the national youth strategy is making emergency contraception (EC) available for these risky population. However, despite its availability in many countries, EC has failed to have the desired impact on unintended pregnancy rates and its utilization is limited in colleges and universities. The objective of this study was to assess factors associated with utilization of emergency contraception among female students in Mizan-Tepi University (MTU), south west Ethiopia.

**Methods:**

A cross-sectional, institution based study was conducted from March 10–30, 2014. Multistage sampling technique was used to select the participants for the quantitative method whereas; purposive and volunteer sampling techniques were used for the qualitative study. Quantitative data were cleaned, coded and entered into Epi-data 3.1 and analyzed using SPSS version 20:00. Binary and multiple logistic regression analysis were done to determine the association between the use of EC and the predicator variables. Data from focus group discussion were transcribed and translated to English then coded, and categorized into similar themes.

**Result:**

A total of 489 female students were participated in the quantitative study making a response rate of 90.6 %. The finding shows that 46.3 % of them have used EC following unprotected sex. Female students’ knowledge about EC [AOR: 3.24; 95 % CI 1.32, 7.98], age at first sexual intercourse (i.e. ≥20 years) [AOR: 4.04; 95 % CI 1.72, 9.52], history of pregnancy [AOR: 3.12; 95 % CI 1.34, 7.24] and previous use of regular contraceptives [AOR: 5.01; 95 % CI 2.23, 11.27] were found to be significant predictors of EC utilization. In the focused group discussion, a total of 32 female students were participated and the result shows that lack of knowledge about EC and fear of being seen by others (information disclosure) were reported as main factors for not using EC.

**Conclusion:**

The study shows that the level of EC use was low. Female students’ level of knowledge about EC, age at first sexual intercourse, previous use of regular contraceptives and history of pregnancy were major predictors of EC utilization. Therefore, designing strategies to enhance EC utilization by increasing female students’ level of awareness on EC is recommended.

**Electronic supplementary material:**

The online version of this article (doi:10.1186/s13104-015-1812-6) contains supplementary material, which is available to authorized users.

## Background

Emergency contraception also called: “post coital contraception”, or “second chance” is a type of modern contraception which is used after unprotected sexual intercourse, following sexual abuse, misuse of regular contraception or non-use of contraception [1]. There are two types of emergency contraception namely Emergency Contraceptive Pills (ECPs) and Intrauterine Contraceptive Devices (IUCD) [2]. The pills include combined emergency contraceptive pills containing estrogen and progestin, and a progestin only pill, which contain only progesterone. If used correctly, all types of ECPs can decrease the risk of unintended pregnancy by more than 75 % which in turn helps to reduce unintended pregnancy and unsafe abortion [2].

Globally, there are about 210 Million pregnancies each year, some 80 million of these are unintended, and one in ten of these pregnancies ends in an unsafe abortion. An estimated 529,000 girls and women die from pregnancy-related causes each year worldwide, of which 13 % are due to unsafe abortion. It’s estimated that two in five unsafe abortions occur among women under age 25 and about one in seven women who have unsafe abortions are under 20 [3, 4]. In developing world, about 56 % of all abortions are unsafe compared to the developed world (6 %) and nearly all unsafe abortions (98 %) occur in developing countries [4]. In Africa, among the annual number of induced abortions (6.4 million), only 3 % are performed under safe conditions. Eastern African countries contributed nearly 39.1 % (2.5 million) of all induced abortions occurred in Africa in 2008, a higher proportion than in any other regions of the continent [5].

Ethiopia is one of the countries with high maternal mortality rate (676 per 100,000 live births), which is responsible for 30 % of all deaths to women of age 15–49. It is estimated that 34 % of all women are either mothers or are pregnant with their first child by age 19 [6]. In 2008, of all pregnancies, about 42 % were unintended, and an estimated 382,500 induced abortions were performed which equates to an annual rate of 23 abortions per 1000 women [7]. In a nationwide hospital based survey of unsafe abortions conducted in nine administrative regions of Ethiopia, unintended pregnancy accounted for 49.1 % of the cases, and non-use of contraceptive methods contributed to 78 % of the pregnancies [8].

Adolescents and women who are not married have less access to reproductive health information and services, and are often highly vulnerable to unintended pregnancies which usually ended up with safe or unsafe abortion as a result of some forms of sexual coercion and violence [9]. University students fall under sexually active age group and form a high-risk group for unintended pregnancy because a large percentage of them engage in sporadic pre- marital sex, which could be prevented by using EC [10]. Different studies have shown that level of knowledge and practices of EC among higher level institution female students is limited [11–14].

In Ethiopia, most of university students travel far from their home for higher education studies, and they are out of care and protection of their parents and families, which makes them more vulnerable to unprotected and accidental sex which leads to unintended pregnancy. Unintended pregnancy either ends with unsafe abortion or early child bearing, it has a negative impact on the educational progress, future careers and even social interaction of female students by forcing them to drop out of school. On the other hand, this segment of the population is tomorrow’s generation in pipeline to take over the responsibilities of socioeconomic development of the country. Thus, they should be protected from unintended pregnancy that could have been considerably prevented by emergency contraception. However, studies conducted on KAP of EC in different Ethiopian universities revealed that there is higher rate of unintended pregnancy with lower level of knowledge, unfavorable attitude towards EC and fewer number of EC practices among female university students [15–18].

Emergency contraception was made available in Ethiopia since 2004. Currently, combined estrogen and progesterone containing pills (YUZPE), 0.75 mg levongestrel/postiner and IUCD are the available EC regiments in the country. As EC has not been included in Ethiopia’s demographic and health survey, no national data exist on EC users. However, in a survey conducted on evaluation of EC mainstreaming campaign, among 3996 EC users in five of the most populated regions of Ethiopia, it has been shown that 41 % of EC users were married, with 47 % were between age group of 20–24 and young women under age 19 comprised 20 % [19].

In one of the studies conducted on knowledge, attitude and practice (KAP) of EC among 660 female students in Adama University, Ethiopia, 84 (27.2 %) had good knowledge about EC, about 415 (62.9 %) had favorable attitude towards the use of EC and from those who are sexually active, 31 (15.9 %) had ever used EC [15]. In another study among 407 female college students in Arbaminch town of Ethiopia, unwanted pregnancy was found to be 51.4 %; about 89 (21.9 %) had good knowledge about EC, about 50 % had favorable attitude towards the use of EC and only 11 (7.9 %) of sexually active students had ever used EC [17].

Several studies were carried out on KAP of EC, however, little is known about the extent of factors associated with EC utilization among higher institutions female students in Ethiopia and in addition to this, no published research has been conducted in the study area. Therefore, the aim of this study is to assess factors associated with utilization of EC among female students in MTU.

## Methods

An institution based cross sectional study was conducted on regular program female students of Mizan-Tepi University from March 10 to 30, 2014 with the objective of assessing factors associated with utilization of emergency contraception. MTU is located 561 km away from the capital city of the country (Addis Ababa) to the south-west of Ethiopia. It is among the emerging 13 new universities established in the country in the last decades. According to the registrar office of the University, MTU has a total of 34 departments under 6 colleges and in the academic year 2013–2014, a total of 8652 students were registered, among which 6174 were in regular program and 1988 were females.

For the quantitative study, the sample size was calculated using a single population proportion formula with confidence interval of 95 % and margin of error 5 %. In the calculation, the prevalence of EC among sexually active female students was considered to be 24.2 % [20]. Then, because the source population (N) is less than 10,000 using the finite population correction formula, the sample size was recalculated and along with 10 % non-response rate and considering the design effect of 2, the final sample size was 540.

As to the sampling technique, a multistage sampling technique method was used; where first, 17 departments were selected from the 34 departments by lottery method, and then the respective sample was allocated to each department proportionally based on the female students’ population in each department. Finally, the study units were selected from each department using simple random sampling technique considering the list of female students as a sample frame.

For the qualitative study, four FGDs were organized, each consisting of eight participants. The participants were selected through volunteer and purposive sampling techniques. Female students who had information on EC practice and related experience were invited voluntarily and members of gender club and female one to five group leaders (locally known as ‘*ande le amest amerar’* in Amharic language) were selected purposively. The rationale for selecting these groups is the fact that they had a chance of attending frequent meetings and had active participation in the teaching learning process and other related topics like sexual and reproductive health issues with other female students in the campus, and assumed to have more insight and rich information on the subject matter.

The quantitative data were collected using pre-tested, structured self-administered questionnaires, and the qualitative part was collected through FGD. The questionnaire was developed after thorough review of various literatures relevant to the study and prepared in English language. It constitutes information about socio-demographic variables, sexual and reproductive history, knowledge, attitude and practice of EC. Before the actual data collection, the questionnaire was pre-tested on 5 % (27 female students) in Aman Poly Technic College which is 10 and 40 km far from Mizan and Tepi towns respectively. The instrument was tested for reliability and validity and accordingly, the cronbach alpha coefficient was found to be 0.84. The questionnaire was distributed to the students by five Graduate Assistants working in Mizan Aman Health Sciences College, and the data were collected while students were in class rooms and the instructors cooperated with data facilitators in disseminating the questionnaire. On completion, the questionnaires were placed in sealed boxes by the participants. Finally, the filled questionnaire was checked for completeness and consistency of the data by the principal investigator on daily basis.

For the qualitative study, a focused group discussion guide was prepared and used to explore additional ideas from the participants. The discussion guide consists of issues about unprotected sex, unintended pregnancy, EC practice and factors that affect its utilization among female students. A total of four FGD were conducted; two for each group separately (i.e. female students who are members of gender club and one to five group leaders and female students who had information on EC practice and related experience). Each FGD consisted of eight participants and took an average of one to one and half hour. Two persons were assigned for note taking and tape recordings while the principal investigator facilitated the discussion for each FGD.

Female students’ knowledge about EC was measured using eight multiple-choice items. Each correct answer awarded one point, and so there were a total of 8 points for the eight items. For questions that consist of more than one correct answer, respondents who have identified at least one possible response were given one point. Based on their cumulative result, those respondents who scored above four out of eight knowledge assessing items were assigned as having “good knowledge” on EC and those who scored four and below were regarded as having “poor knowledge”. Students’ attitude towards EC was assessed using eight items rated on a five-point Likert scale as (1) strongly agree, (2) agree, (3) not sure, (4) disagree and (5) strongly disagree. For the purpose of ease of analysis, attitude of female students towards the utilization of EC items were condensed into three categories as “agree”, “disagree” and “not sure”. Furthermore, the attitude measurement Likert scale was summarized as “favorable attitude” (those who scored above the mean on attitude items), and “unfavorable attitude” (those who scored the mean or below mean to attitude measuring items).

Before conducting the study, it was submitted to Jimma University and letter of ethical clearance approval was obtained from Jimma University, College of Public Health and Medical Sciences, Ethical Review Board (ERB). The purpose of the study was explained to the study participants and privacy and confidentiality was ensured. Prior to data collection, informed verbal consent was obtained from the study participants. While obtaining consent from each participant, information related to publishing the study finding and responses of both quantitative and qualitative data were addressed. Specifically for the qualitative study, consent to publish all the quoted information used in this article was also obtained from FGD participants. For three of the study participants whose age was 17 years and involved in the quantitative study, parental informed consent was obtained. The respondents’ right to refuse or withdraw from participating in the study was fully acknowledged.

The collected quantitative data was cleaned, coded and entered into Epi-data 3.1 software, and then exported to Statistical Package for Social Sciences (SPSS) version 20:00 for analysis. First, descriptive analysis was carried out for each variable. Next, bivariate analysis was done to identify the association between the independent and the outcome variables to select the candidate variables for the multivariable analysis. Accordingly, those variables with a p value <0.25 in bivariate analysis (i.e. respondents age, year of study, field of study, marital status, age at first sexual intercourse, history of pregnancy, experience of regular contraceptives, knowledge about EC and attitude towards EC) were selected for multivariate logistic regression, and then those variables with a p value <0.05 were considered to be statistically significant in multivariate analysis.

The audio taped data from focus group discussion was transcribed and translated to English and then the written data were coded by two researchers. Then, subsequent pattern formation and thematization were done from the coded data. Finally, some quotes that could explain the context of factors affecting EC utilization were identified and presented in the respondents’ own words to give more insight.

## Result

### Qualitative study

In the qualitative study, a total of 32 female students from different fields of study, ethnic and religious groups were participated in four FGDs, each consisting of 8 discussants which took an average of one to one and half hour. The mean age was 22.4 (±1.2) years, the youngest being 18 and the oldest 28. Knowledge about EC, attitude towards EC, and fear of information disclosure were identified as major themes from the discussion. As an outliers religious outlook and economical problem (i.e. cost of EC pills), were mentioned by two FGD participants from volunteer groups and categorized under minor theme. But these two ideas were opposed by the rest of the discussants.

### Quantitative study

#### Socio demographic characteristics

A total of 489 female students participated in the study, making a response rate of 90.6 %. The mean age of the respondents was 20.7 (±1.7) years, the youngest being 17 and the oldest 31 years old. More than half (51.5 %) of the respondents were Orthodox Christian by religion followed by Protestants 141 (28.8 %) and Muslim 67 (13.7 %). Regarding their ethnicity, Amhara 193 (39.5 %); Oromo, 174 (35.6 %); followed by Tigres 46 (9.4 %) and most of the students (90.5 %) were unmarried (Table 1).

**Table 1:** Socio-demographic characteristics of female students, Mizan-Tepi University, south west Ethiopia, March, 2014 (n = 489)

Variables	Number	Percent
Age category		
15–19	154	31.5
20–24	323	66.1
25–29	9	1.8
≥30	3	0.6
Year of study		
First year	134	27.4
Second year	126	25.8
Third year	169	34.6
Fourth year	52	10.6
Fifth year	8	1.6
Field of study		
None health sciences	431	88.1
Health sciences	58	11.9
Marital status		
Single	448	91.6
Married	38	7.8
Divorced	3	0.6
Religion		
Orthodox	250	51.2
Protestant	141	28.8
Muslim	67	13.7
Catholic	21	4.3
Others^a^	10	2.0
Ethnicity		
Amhara	193	39.5
Oromo	174	35.6
Tigrie	46	9.4
Wolita	29	5.9
Gurage	25	5.1
Others^b^	22	4.5

^a^Other ethnic group refers to Keffa, Dawro, Silte, Sidama and Bench

^b^Other religious group refers to Adventist and Jehovah

#### Sexual and reproductive health history

Out of the total 489 female students, 188 (38.4 %) ever had sex since they entered in the campus of which 123 (65.4 %) had unprotected sex, 81 (43.1 %) had been pregnant and the majority 69 (85.2 %) experienced unintended pregnancies (Table 2).

**Table 2:** Sexual and reproductive history of female students, Mizan-Tepi University, South west Ethiopia, March, 2014

Variables	Number	Percent
Ever had sex since in the campus (n = 489)		
Yes	188	38.4
No	301	61.6
Age at first sex (n = 188)		
15–19	122	64.8
≥20	66	35.1
Unprotected sex^a^ (n = 188)		
Yes	123	65.4
No	65	34.6
History of pregnancy (n = 188)		
Yes	81	43.1
No	107	56.9
Unintended pregnancy (n = 81)		
Yes	69	85.2
No	12	14.8

^a^Unprotected against pregnancy

#### Knowledge, attitude and practice of emergency contraception

Among female students who heard about EC 332 (67.8 %), only 94 (28.3 %) correctly identified time of administration of the method, 54 (16.3 %) the recommended doses, and 49 (14.8 %) the recommended number of doses and the time interval between the doses. The finding of the FGD also supports this idea. A 20 years old second year student said *“……most of the students have no clear information about EC, even we ourselves don’t know how and when to take the EC”.* Furthermore, the knowledge summary index about EC also shows that the majority, 238 (71.7 %) of the respondents had poor knowledge on EC, and this finding is also strengthened by the qualitative study, “……….it was 6 months ago; a female student came to gender club in order to get counseling service after she confirms her pregnancy. When I asked her why she didn’t take EC, she responded that even she don’t know about it at all*.”* Said a 22 year’s old, third year, student and member of gender club (Table 3).

**Table 3:** Knowledge about emergency contraception among female students, Mizan-Tepi University, south west Ethiopia, March, 2014 (n = 332)

Knowledge assessment items	Number	Percent
Where do you think emergency contraception could be obtained		
Pharmacy/Health facility	87	26.2
Any shops	143	43.1
I don’t know	102	30.7
Which one of these drugs can be used for emergency contraception		
Combined oral contraceptive	98	29.5
Progesterone only pills & IUCD	90	27.1
Anti-biotic like ampicillin	34	10.2
I don’t know	110	33.2
When taken early, emergency contraception prevent sexually transmitted infections		
Yes	158	47.5
No	51	15.4
I don’t know	123	37.1
Situation(s) that emergency contraception should be taken		
If condom ruptured during intercourse	109	32.8
When there is a missed pill	78	23.4
When forced to have sex/rape	140	42.1
When there is failure of contraception	169	50.9
I don’t know	145	43.6
The recommended maximum time limit to take emergency contraception pills		
Within 24 h after sex	120	36.1
Within 72 h after sex	94	28.3
Within 5 days after sex	42	12.7
I don’t know	76	22.9
Effectiveness of emergency contraception pills in preventing pregnancy		
Highly effective (>95 %)	52	15.7
Effective (75–89 %)	68	20.5
Less effective (<10 %)	29	8.7
Not effective at all	40	12.0
I don’t know	143	43.1
Recommended number of dose of emergency contraception pills		
One dose	50	15.1
Two doses	54	16.3
Three doses	35	10.5
I don’t know	193	58.1
Recommended time between the doses of emergency contraception pills		
Twelve hours apart	49	14.8
Twenty-four hours apart	45	13.5
Seventy-two hours apart	50	15.1
I don’t know	188	56.6
Knowledge of EC (Summary index)		
Good knowledge	80	24.1
Poor knowledge	252	75.9

With regard to attitudes towards EC, about 246 (50.3 %) of the respondents agreed that EC use may cause infertility in a woman. Supporting this idea, one of the discussants said, sharing the lived experience of one of her colleagues *“*……… her reason for not taking EC was that she had the experience of taking EC once previously and now she feared that she might not be able to give birth in her future life and prefers to give birth instead of taking the pills for the second time even if it happens*.”* Concerning the overall level of female students’ attitudes, more than half (53.2 %) of them had unfavorable attitude towards EC (Table 4).

**Table 4:** Attitude towards emergency contraception among female students, Mizan-Tepi University, south west Ethiopia, March, 2014 (n = 489)

Attitude assessment items	Disagree		Not sure		Agree	
	No	%	No	%	No	%
Provision of Emergency contraception after an episode of un-protected sex can prevent unwanted pregnancy	131	26.8	282	57.7	76	15.5
All females have the right to access emergency contraception	96	19.6	161	33.0	232	47.4
Emergency contraception promotes promiscuity	255	52.1	86	17.6	148	30.3
Emergency contraception may hurt the baby in case it does not work	72	14.7	164	33.6	253	51.7
Emergency contraception is one way of abortion	106	21.7	242	49.5	141	28.8
It is sinful act to use emergency contraception	84	17.2	273	55.9	132	26.9
Emergency contraception use may cause infertility in a woman	135	27.6	108	22.1	246	50.3
Emergency contraception will affect ongoing regular methods of contraception negatively	101	20.7	226	46.2	162	33.1

Attitude of EC (Summary index)	No	%
Favorable attitude	229	46.8
Unfavorable attitude	260	53.2

**Fig. 1:**
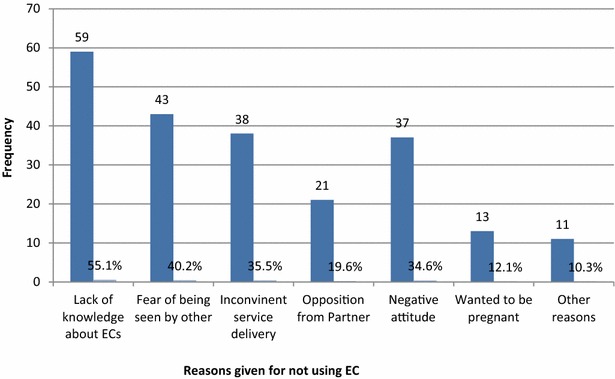
Reasons for non-users of emergency contraception, among female students with unprotected sex, Mizan-Tepi university, south west Ethiopia, March, 2014 (n = 66)

Regarding the utilization of EC, among those who had ever sex since in the campus, only 68 (36.2 %) had ever used EC and it was 57 (46.3 %) among those who had unprotected sex and female friends were the major source of information for EC users (Table 5). Among sexually active respondents who did not use EC, lack of knowledge about EC (58.1 %) and fear of being seen by others (43.9 %) were the main reasons (Fig. 1). Strengthening this finding, one of the discussant shared the lived experience of her friend and said “…..she feared to go to pharmacy and buy EC pills and 5 days elapsed after unprotected sex and finally, she told me about the case, then, I brought to her EC pills and then she took the pills, but at the end she became pregnant and she underwent medical abortion at private clinic.”

**Table 5:** Emergency contraception utilization among female students of Mizan-Tepi University, south west Ethiopia, March, 2014

Variables	Number	Percent
Used EC among those who had sex since in the campus (n = 188)		
Yes	68	36.2
No	120	63.8
Used EC among those who had unprotected sex (n = 123)		
Yes	57	46.3
No	66	53.7
Types of EC used (n = 68)		
ECPs	66	97.1
IUCD	2	2.9
Source of information (n = 68)^a^		
Female friends	36	52.9
Sexual partner	30	44.1
Mass media	12	17.6
Health professional/facility	10	14.7
Web pages	7	10.2
Other sources	4	4.4

^a^Indicates that multiple response is possible for that item

#### Predictors of emergency contraception utilization

The multivariate logistic regression analysis shows that female students who had first sexual intercourse at age 20 and above were four times more likely to use EC as compared to those who had their first sexual intercourse at younger age (15–19 years), [AOR 4.048; 95 % CI 1.721, 9.524]. Respondents who had history of pregnancy were 3 times more likely to use EC than those with no exposure of pregnancy [AOR: 3.122; 95 % CI 1.346, 7.240]. Similarly, students who had good knowledge on EC were 3.2 times more likely to use EC than those female students who were not knowledgeable about EC [AOR: 3.248; 95 % CI 1.320, 7.988]. The result also showed that respondents who had experience of other forms of regular contraceptive use were 5 times more likely to use EC than those who didn’t use other forms of regular contraceptives [AOR: 5.019; 95 % CI 2.234, 11.274] (Table 6).

**Table 6:** Predictors of emergency contraception utilization among female students, Mizan-Tepi University, south west Ethiopia, March, 2014 (n = 188)

Variables	Used EC		Odds Ratio	
	Yes	No	COR (95 % CI)	AOR (95 % CI)
	N (%)	N (%)		
Age				
≥20 years	45 (66.2 %)	61 (50.8 %)	1.89 (1.02, 3.50)	2.12 (.45, 9.98)
15–19 years	23 (33.8 %)	59 (49.2 %)	1	1
Year of study				
Year II and above	54 (79.4 %)	77 (64.2 %)	2.15 (1.07, 4.32)	.60 (.13, 2.79)
Year I	14 (20.6 %)	43 (35.8 %)	1	1
Field of study				
Health sciences	21 (30.9 %)	25 (20.8 %)	1.69 (.86, 3.34)	.52 (.18, 1.48)
None Health sciences	47 (69.1 %)	95 (79.2 %)	1	1
Marital status				
Ever married	22 (32.4 %)	10 (8.3 %)	5.26 (2.31,11.98)	2.90 (.95, 8.78)
Singles	46 (67.6 %)	110 (91.7 %)	1	1
Age at first sexual intercourse				
≥20 years	43 (63.2 %)	23 (19.2 %)	7.25 (3.71,14.18)	4.04 (1.72, 9.52) *
15–19 years	25 (36.8 %)	97(80.8 %)	1	1
History of pregnancy				
Yes	47 (69.1 %)	34 (28.3 %)	5.66 (2.95,10.84)	3.12 (1.34, 7.24)*
No	21 (30.9 %)	86 (71.7 %)	1	1
Ever use regular contraceptives				
Yes	46 (67.6 %)	31 (25.8 %)	6.00 (3.12,11.52)	5.01 (2.23,11.27)**
No	22 (32.4 %)	89 (74.2 %)	1	1
Knowledge on EC				
Good knowledge	53 (77.9 %)	29 (24.2 %)	11.08 (5.45,22.53)	3.24 (1.32, 7.98)*
Poor knowledge	15 (22.1 %)	91 (75.8 %)	1	1
Attitude towards EC				
Favorable attitude	51 (75.0 %)	70 (58.3 %)	2.14 (1.11,4.13)	1.95 (.80, 4.75)
Un Favorable attitude	17 (25.0 %)	50 (41.7 %)	1	1

* P value of <0.05 and ** P value of <0.001

## Discussion

The finding of the study shows that 24.1 and 46.8 % of female students were found to have good knowledge and favorable attitude towards EC respectively. This finding is consistent with the studies conducted in Arbaminch and Jimma [17, 18], but much lower than that of Cameroon and Nepal [21, 22]. This difference might be attributed to the differences in provision of sexual and reproductive health education at schools and higher learning institutions as well better practice of open and free discussion on sex and sexuality among female students in these countries.

With respect to the use of EC, among those who had ever sex since entered in the campus, only 68 (36.2 %) had ever used EC and it was 57 (46.3 %) among those who had unprotected sex, the discrepancy occurred because 11 students used EC while having protected sex and the possible explanations for this could be either they might prefer to use dual contraceptive method or might be due to missed pills of regular contraceptive (i.e. as backup method). This finding is inconsistent with the study conducted in Adama University [16]. The possible explanation for a higher EC practice observed in this study might be related to differences in the sample size between the two studies and increase in the level of awareness as the time of the study goes on might also be another possible explanation.

As to the predicators of use of EC, the multivariate regression analysis showed that good knowledge about EC was significantly associated with the use of emergency contraceptives. This is consistent with studies done in Arbaminch and Cameroon [17, 21].

In line with the finding of study conducted in Uganda, EC utilization was found to be higher among female students with history of pregnancy than their counterparts [Asinja K: knowledge, use and attitudes towards emergency contraception among female students of teacher’s Colleges in Mid-western Uganda, submitted to the School of Graduate of Makerere University]. The lesson learned from previous pregnancy might be the possible explanation for higher practice of EC among female students with history of pregnancy.

Female students who started first sexual intercourse at age ≥20 years were found to be more likely to use EC than those who started first sexual intercourse at an earlier age ≤19 years. This finding is similar with a study done in Adama University [16]. This could be due to better exposure to information or increased awareness about EC, maturity, and experiences of the consequences of unintended pregnancies held by these girls as they started sex at older age when compared to those who had first sex at earlier ages.

The other finding of this study in multivariate regression analysis is that previous experience of regular contraceptives had statistically significant association with EC utilization. Female students who had used regular contraceptive previously were more likely to use EC when compared to those who had no previous experience of regular contraceptive methods. This finding is consistent with the studies done in Adama University and Makerere University [16]. This might be related to the experience of using different family planning services including EC by those who have the exposure than those who didn’t have. Higher frequency of sex among female students who had used regular contraception could also be the other possible explanation for higher practice of EC.

In general, higher education female students are exposed to many sexual and reproductive health problems among which unintended pregnancy is one of them, which could be prevented by early use of EC. However, as shown in this study, correct knowledge and utilization of EC among MTU female students is limited. Therefore, this type of study will help strategy and policy makers in developing appropriate evidence-based strategies and curricula in higher institutions to prevent unintended pregnancy. The study applied a mixed method to strengthen and support the quantitative data. As a limitation, this study cannot ascertain cause and effect relationship since it is a cross-sectional type, social desirability bias cannot be totally eliminated as the study touches sensitive issues that might lead to under reporting, lack of detailed and more in-depth qualitative study and problems related with translation of discussants’ original words could also be potential limitation of the study (Additional file 1).

## Conclusion

The study finding shows that there is relatively higher proportion of EC users than other studies indicate. However, still, it can be considered very low with high percentage of unintended pregnancy among sexually active students. The study finding also showed that level of knowledge of EC, time of first sexual intercourse, previous use of regular contraceptives and history of being pregnant were the major predictors for EC utilization among female students of MTU. Additionally, it was indicated in the result that female students’ knowledge about the general features of EC is low and misinformation is high. Therefore, it is recommended that strategy and policy makers should develop appropriate evidence-based strategies and curricula in higher institutions to prevent unintended pregnancy and to promote the need based use of EC. Thus, designing strategies towards awareness creation and attitude changing activities about EC through provision of different regular health information and communication programs is implicated. Working in collaboration with governmental and nongovernmental organizations whose focus is to address the need of reproductive health services, with particular emphasis to family planning/EC, is therefore recommended for enhancing (scaling up) EC utilization among female students. Finally, we recommend a separate, detailed and more in-depth qualitative study on factors associated with EC utilization and further research on the level and the type of forced sexual intercourse among female University students is also recommended, which was the third major reason for unintended pregnancy in this study (Additional file 2).

## Additional files

10.1186/s13104-015-1812-6 COREQ checklist for focus group.
10.1186/s13104-015-1812-6 English version of the questionnaire.

## Abbreviations

EC: emergency contraception
ECPs: emergency contraceptive pills
ERB: ethical review board
FGD: focused GROUP discussion
IUCD: intra uterine contraceptive device
KAP: knowledge, attitude, and practice
MTU: Mizan-Tepi university
SPSS: statistical package for social science
