# Supplementary material for: Factors associated with utilization of emergency contraception among female students in Mizan-Tepi University, South West Ethiopia
Source: BMC Res Notes. 2015 Dec 24;8:817. doi: 10.1186/s13104-015-1812-6 (PMC4691018; doi:10.1186/s13104-015-1812-6)
Supplement: Supplementary file 1 — 10.1186/s13104-015-1812-6 COREQ checklist for focus group. [file 13104_2015_1812_MOESM1_ESM.rtf]

COREQ CHECKLIST FOR FOCUS GROUP 
NO.	ITEM	DESCRIPTION	
1.		 Interviewer/facilitator 	The corresponding author facilitated the focus group 	
2.		Credentials 	Bisrat Zeleke Shiferaw (BSc.N, MSc.in maternity health nursing) 1 Bosena Tebeje Gashaw (BSc.N, MSC in reproductive health, Assistant Professor.) 2 Fekadu Yadassa Tesso (BSc.N, MSc. in maternity and reproductive health nursing) 3 
	
3.		Occupation 	Researcher occupation at the time of the study 
Bisrat Zeleke Shiferaw: - Lecturer- Department of midwifery, Mizan-Aman health sciences college.	
1.			Bosena Tebeje Gashaw: - Assistant Professor of Maternal and Reproductive Health, college of Public Health and Medical Sciences, Department of Nursing and Midwifery, Jimma university.	
1.			Fekadu Yadassa Tesso: - Senior lecturer, college of Public Health and Medical Sciences, Department of Nursing and Midwifery, Jimma university	
4.		Gender 	Bisrat Zeleke Shiferaw ------ Male 	
1.			Bosena Tebeje Gashaw ------Female	
1.			Fekadu Yadassa Tesso------ Male
	
5.		Experience and training 	Bisrat Zeleke Shiferaw
·	An academic staff of Wolkite University with 5 years of experience in mizan Aman health Sciences College and Wolkite University College of medicine and Health Sciences.
·	One finalized unpublished research on breast self-examination (which is on the way to start the publication process )
·	Two ongoing research on gender based violence and predictors of optimal breast feeding practices.
·	Certified national trainer by Federal ministry of health on PMTCT option B+ and integrated management of neonatal and childhood illness.
Bosena Tebeje Gashaw
·	An academic staff with more than 25 years of experience in college of Public Health and Medical Sciences, Jimma University.
·	Nine published researches on different journals (both as a principal and co-investigator)
·	Majorly involved on preparations of four Module, book and Lecture Note for health sciences students
Fekadu Yadassa Tesso
·	An academic staff with more than 20 years of experience in college of Public Health and Medical Sciences, Jimma University.
·	Unpublished two qualitative researches with Swedish professors and Norwegian professors. 
·	Experienced data collector of qualitative research	
6.		Relationship established 	There was no established relationship between the researchers and their participants prior to study commencement, except the communication established for the purpose of explaining the aim of doing the research and gaining informed consent prior to the data collection.	
7.		Participant knowledge of the interviewer
	The researcher state their assumptions and interests in the research topic and also the purpose of doing the research was explained to the study participants prior to the data collection.
	
8.		Interviewer characteristics 	Facilitator characteristics on reasons and interests in the research topic were reported 	

9.		
Methodological orientation and Theory
	The study applied a mixed method research design (a concurrent triangulation design) where the focus is confirming, cross validating or corroborating the finding. 	
10.		Sampling 	Participants were selected by using purposive and volunteer sampling techniques.	
11.		Method of approach 	The focus group was conducted by face-to-face approach (method).	
12.		Sample size 	A total of 32 participants were participated in 4 organized focus groups each containing 8 discussants.	
13.		Non-participation 	All participants were participated and none of them refused to participate or dropped out.	
14.		Setting of data collection 	The data was collected in the campus (study area)	
15.		Presence of non-participants 	During the discussion the facilitator, one note taker and one tape recording facilitator were present besides the participants.	
16.		Description of sample 	The participants were from different age group, fields of study ethnic and religious groups.	
17.		Interview guide 	The discussion questions and prompts were tested prior to the study on college students. The questions and prompts used for the actual data collection were provided to the participants. 	
18.		Repeat interviews 	Repeat interviews were not carried out.	
19.		Audio/visual recording 	The research also used audio recording to collect the data.	
20.		Field notes 	Field notes were also taken during focus group to record the participants' words and to maintain the contextual details and non-verbal expressions.	
21.		Duration 	One FGD took an average of one to one and a half hour.	
22.		Data saturation 	Participants were invited until no new relevant knowledge was being obtained from new participants (i.e. Data saturation was discussed) 	
23.		Transcripts returned 	Transcripts were returned to participants to check their own transcript for accuracy, comment and correction.	
24.		Number of data coders 	Data was coded by two researchers.	
25.		Description of the coding tree 	The data coding process is described 	
26.		Derivation of themes 	Themes were derived from the collected data	
27.		Software 	Software was not used to manage the data	
28.		Participant checking 	Feedback on the research ﬁndings was obtained from participants 	
29.		Quotations presented 	Quotes that could explain the context of factors affecting EC utilization were identified and presented in the respondents' own words.	
30.		Data and ﬁndings consistent 	There was consistency between the presented data and the ﬁndings	
31.		Clarity of major themes 	The major themes from the study were clearly presented in the ﬁndings.	
32.		Clarity of minor themes 	Diverse cases (outliers) from the major theme were described or in the ﬁndings.	
