# Supplementary material for: Factors associated with utilization of emergency contraception among female students in Mizan-Tepi University, South West Ethiopia
Source: BMC Res Notes. 2015 Dec 24;8:817. doi: 10.1186/s13104-015-1812-6 (PMC4691018; doi:10.1186/s13104-015-1812-6)
Supplement: Supplementary file 2 — 10.1186/s13104-015-1812-6 English version of the questionnaire. [file 13104_2015_1812_MOESM2_ESM.rtf]

QUESTIONNAIRE
Introduction 
Dear participants, this questionnaire is prepared to asses factors associated with utilization of Emergency Contraception among female students in Mizan-Tepi University. It contains five sections namely; socio-demographic section, sexual and reproductive history section, Emergency Contraception knowledge assessment section, Emergency Contraception attitude assessment section and Emergency Contraception practice assessment. 
The ultimate purpose of this study is to identify those factors associated with utilization of Emergency Contraception among female students of Mizan-Tepi University. The findings of this study will help to develop appropriate strategies for tackling the problem. To attain this aim your honest and genuine participation is very important and highly appreciable. 
We (the researchers) therefore, kindly request you to fill this questionnaire as accurately and carefully as possible. Please, be assured that all the information gathered will be kept strictly confidential and you do not need to write your name or any special identification that might disclose who you are, on any of the questionnaire page. 
Thanks for your honest and genuine participation!


SECTION I- SOCIO- DEMOGRAPHIC CHARACTERISTICS OF THE RESPONDENTS
Dear participants, this section assess some of your socio-demographic feature like age, marital status and so on. It consists of six items. For close ended questions, please encircle your response among the provided alternatives. If your response is not included among the provided alternatives, write your responses on the space provided under “if other specify” option. For open ended items write your response precisely & clearly on the space provided for each item.
ITEM CODE	QUESTION	POSSIBLE  RESPONSES	SKIP	
SD101	How old are you? (Age in completed years)	
       ---------------------------		
SD102	Which year of study are you attending now?  	1.	First year
2.	Second year
3.	Third year
4.	Fourth year
5.	Fifth year
		
SD103	Which field of study are you attending now?  (your field of study)	
  --------------------------------------		
SD104	What is your current Marital status?   
	1.	Single 
2.	Married 
3.	Divorced                                                                
4.	Widowed                               
		
SD105	Which religious group are you following?   	1.	Orthodox 
2.	Protestant
3.	Muslim    
4.	Catholic
99.	If Other,( specify )--------------------------------------
		
SD106	What is your ethnic group?     
 	1.	Amhara 
2.	Oromo
3.	Tigrie 
4.	Wolita 
5.	Gurage
99.	If other (specify) ---------------------------------------		


SECTION - II - RESPONDENTS SEXUAL AND REPRODUCTIVE HISTORY 
Dear participants, section two assess some of your sexual and reproductive history and it consists six items. For close ended questions, please encircle your response among the provided alternatives. If your response is not included among the provided alternatives write your responses on the space provided under “if other specify” option. For open ended items write your response on the precisely & clearly on the space provided for each item. Please, ensure that you are following the skip rule column for items that needs to be jumped while filling the each items.
ITEM CODE	QUESTION	POSSIBLE  RESPONSES	SKIP	
SR201	Have you ever had sex since you joined this campus? 
	1.	Yes 
2.	No	
If  No         KG301	
SR202	If your response is YES for item code.  SR201, What was your age during the first sexual intercourse?
	
        -------------------------		
SR203	Have you ever had Unprotected sexual intercourse since you joined this campus? 
	1.	Yes  
2.	No 	If  No         SR206	
SR204	If your response is YES for item code.  SR203 did you get pregnant?	1.	Yes
2.	No 	If  No         SR206	
SR205	If your response is YES for item code.  SR204 was it Unintended pregnancy 
	1.	Yes 
2.	No		
SR206	Have you ever used any regular contraceptive previously? 
	1.	Yes 
2.	No		

SECTION - III - RESPONDENTS KNOWLEDGE ASSESSMENT QUESTIONS ON EMERGENCY CONTRACEPTION
Dear participants, this section assess your knowledge about emergency contraception. It consists eight items. For each item the possible alternatives are provided please, encircle your response among the provided alternatives. For items that might have more than one responses you can encircle multiple possible alternatives. 
ITEM CODE	QUESTION	POSSIBLE  RESPONSES	SKIP	

KG301	
Where do you think emergency contraception could be obtained?
        	
1.	 Pharmacy/ Health facility
2.	Any shops
77.I don't know
		
KG302	Which one of these drugs can be used for emergency contraception?	1.	Combined oral contraceptive 
2.	Progesterone only pills & IUCD
3.	Anti-biotic like ampicillin
77.I don't know		
KG303	When taken early, emergency contraception prevent sexually transmitted infections  
	1.	Yes
2.	 No 
77.I don't know		
GK304	Situation(s) that emergency contraception should be taken (more than one answer could be possible)
           	1.	If condom ruptured during intercourse
2.	When there is a missed pill
3.	When forced to have sex/raped
4.	When there is failure of contraception
77.I don't know
		
GK305	What is the maximum recommended time limit to take emergency contraception Pills?
	1.	Within 24 hours after sex
2.	Within 72 hours after sex
3.	Within 5 days after sex
77.I don't know
		
KG306	How much do you think is the effectiveness of emergency contraception Pills in preventing pregnancy?
      	1.	Highly effective (>95%)
2.	Effective (75-89%)
3.	Less effective (<10%)  
4.	Not effective at all
77.I don't know
		
KG307	What is the recommended number of dose of emergency contraception Pills?

     	1.	One dose
2.	Two doses
3.	Three doses
77.I don't know
		
KG308	What is the recommended time interval between the doses of emergency contraception Pills?
	1.	12 hours apart 
2.	 24 hours apart
3.	72 hours apart
77.I don't know
		

SECTION –IV - RESPONDENTS ATTITUDE ASSESSMENT QUESTIONS TOWARDS EC
Dear participants, this section assess your attitude towards emergency contraception. It consists eight items having five Likert scale alternatives. For each item five Likert scale alternatives are provided from “strongly agree” to “strongly disagree” please, encircle one of your response among the provided alternatives. 
ITEM CODE	QUESTION	POSSIBLE RESPONSES	SKIP	
AT401	Provision of  Emergency contraception after unsafe sexual intercourse can prevent unwanted pregnancy
	1.	Strongly agree 
2.	Agree
3.	Neutral  
4.	Disagree
5.	Strongly Disagree		
AT402	All females have the right to access EC.
	1.	Strongly agree 
2.	Agree
3.	Neutral  
4.	Disagree
5.	Strongly Disagree		
AT403	Emergency contraception promotes promiscuity 
	1.	Strongly agree 
2.	Agree
3.	Neutral  
4.	Disagree
5.	Strongly Disagree		
AT404	 Emergency contraception  may hurt the baby in case it does not work
	1.	Strongly agree 
2.	Agree
3.	Neutral  
4.	Disagree
5.	Strongly Disagree		
AT405	Using Emergency contraception to prevent unintended pregnancy  is one way of abortion 
	1.	Strongly agree 
2.	Agree
3.	Neutral  
4.	Disagree
5.	Strongly Disagree
1.	Stron		
AT406	Using Emergency contraception to prevent unintended pregnancy is a sinful act 
	1.	Strongly agree 
2.	Agree
3.	Neutral  
4.	Disagree
5.	Strongly Disagree		

AT407	
Using Emergency contraception may cause infertility in a woman
	1.	Strongly agree 
2.	Agree
3.	Neutral  
4.	Disagree
5.	Strongly Disagree 		
AT408	Emergency contraception will affect ongoing regular methods of contraception negatively
	1.	Strongly agree 
2.	Agree
3.	Neutral  
4.	Disagree
5.	Strongly Disagree		
SECTION - V - RESPONDENTS EXPERIENCE OF EC 
Dear participants, this section assess your experience of emergency contraception utilization. It consists four items. This section should only be filled by respondents who had sexual contact history since they joined campus. For each item the possible alternatives are provided please, encircle your response among the provided alternatives. If your response is not included among the provided alternatives, write your responses on the space provided under “if other specify” option.  For items that might have more than one response, you can encircle multiple possible alternatives. Please, ensure that you are following the skip rule column for items that needs to be jumped while filling the each items. 
ITEM CODE	QUESTION	POSSIBLE RESPONSES	SKIP	
EP501	Have you ever used emergency contraceptive since you joined this campus? 
	1.	Yes   
2.	No	If No        EP504	
EP502	If your response is YES for item code EP501 Which methods did you used? 
	1.	Emergency contraception Pills
2.	IUCD		
EP503	If your response is YES for item code EP501, where did you get the information? (more than one response could be possible)	1.	Female friend
2.	Sexual partner  
3.	Mass media
4.	Health professional /facility
5.	Web pages 
99.	If other (specify)--------------------------------------------------------		
EP504	If your response is NO for item code EP501 what was the reason for not using EC? (More than one response could be possible) 	1.	Lack of knowledge  about ECs
2.	Fear of being seen by other 
3.	Inconvenient service delivery  
4.	Opposition from partner to use condom 
5.	Negative attitude towards EC
6.	Wanted to be pregnant 
99.	If other(specify)---------------------------------------------------------		
